# Biological control strategy with *Poecilia reticulata* for the reduction of *Aedes* spp. and *Culex* sp. in urban stormwater drains: Experience at *Universidad del Valle*, Cali, Colombia

**DOI:** 10.7705/biomedica.7960

**Published:** 2026-08-20

**Authors:** Olga Lucía Velásquez, Gissel Guzmán, Olga Lucía Cuéllar, Madelaine Piedrahita, Carlos Alberto Reina

**Affiliations:** 1 Subgrupo de Prevención, Vigilancia y Control de las Enfermedades Transmitidas por Vectores, Grupo de Salud Ambiental, Secretaría de Salud del Distrito Especial de Cali, Cali, Colombia Secretaría de Salud del Distrito Especial de Cali Cali Colombia; 2 Subgrupo de Vigilancia y Demografía, Grupo Salud Pública Cali, Secretaría de Salud del Distrito Especial de Cali, Cali, Colombia Secretaría de Salud del Distrito Especial de Cali Cali Colombia; 3 Facultad de Salud, Fundación del Área Andina, Bogotá, D. C., Colombia Fundación del Área Andina Bogotá D. C Colombia

**Keywords:** Poecilia reticulata, Aedes aegypti, *Culex* sp., pest control, biological, Bayes theorem, dengue/epidemiology, arbovirus infections, public health, Poecilia reticulata, Aedes aegypti, *Culex* sp., control biológico de vectores, teorema de Bayes, dengue/epidemiología, infecciones por arbovirus, salud pública

## Abstract

**Introduction.:**

Dengue is one of the most significant arboviral diseases in Latin America and the Caribbean, with *Aedes aegypti* as its primary vector. The elimination of breeding sites is the most effective control strategy; however, the presence of stormwater drains in urban environments poses a challenge.

**Objective.:**

To evaluate the effectiveness of biological control using *Poecilia reticulata* in reducing *Aedes (Aedes aegypti* and *Aedes albopictus)* and *Culex* sp. larvae in urban stormwater drains at *Universidad del Valle,* Cali, Colombia.

**Materials and methods.:**

A quasi-experimental study was conducted with a nine-month follow-up (October 2022 - June 2023). All stormwater drains catch basins at the University were inspected, and 12 catch basins were selected that contained *Aedes (A. aegypti* and *A. albopictus)* and *Culex* sp. mosquito larvae. The number of mosquito larvae by genus, water temperature, and pH were recorded weekly. The intervention involved the introduction and monitoring of *P. reticulata,* with reintroductions as needed. A Bayesian multilevel logistic regression model was used to estimate the intervention effect, adjusting for environmental factors and variability among stormwater drains.

**Results.:**

The intervention group showed a trend toward lower larval presence, although it did not reach statistical significance (ß = -0.90, 95% CI: -2.01 to 0.21). Water temperature was positively associated with larval presence (ß = 0.25, 95% CI: 0.07 to 0.45), while pH showed a negative association (ß = -1.50, 95% CI: -3.06 to -0.06), indicating reduced larval development in more alkaline water.

**Conclusions.:**

Biological control with *P. reticulata* showed potential for reducing larval presence. Temperature and pH were key determinants of larval presence, emphasizing the need to consider environmental factors in vector control strategies.

## Resumen

**Introducción.:**

El dengue es una de las enfermedades arbovirales más importantes en Latinoamérica y el Caribe, y *Aedes aegypti* es su principal vector. La eliminación de criaderos es la estrategia de control más eficaz; sin embargo, la presencia de desagües pluviales en entornos urbanos representa un reto.

**Objetivo.:**

Evaluar la efectividad del control biológico con *Poecilia reticulata* para reducir la población de larvas de *Aedes (A. aegypti* y *A. albopictus)* y *Culex* sp. en desagües pluviales urbanos de la Universidad del Valle, Cali, Colombia.

**Materiales y métodos.:**

Se realizó un estudio cuasiexperimental con un seguimiento de nueve meses (octubre de 2022 a junio de 2023). Se inspeccionaron todos los sumideros de aguas lluvias de la Universidad y se eligieron 12 sumideros que tenían larvas de mosquitos de *Aedes (A. aegypti* and *A. albopictus)* y *Culex* sp. Se registró semanalmente la cantidad de larvas de mosquitos por género, temperatura del agua y pH. La intervención consistió en la introducción y el monitoreo de *P. reticulata,* con reintroducciones según fuera necesario. Se utilizó un modelo bayesiano de regresión logística multinivel para estimar el efecto de la intervención, ajustando los factores ambientales y la variabilidad entre los desagües pluviales.

**Resultados.:**

El grupo de intervención mostró una tendencia hacia una menor presencia de larvas, aunque no alcanzó la significación estadística (ß = -0,90; IC_95%_: -2,01 a 0,21). La temperatura del agua se asoció positivamente con la presencia de larvas (ß = 0,25; IC_95%_: 0,07 a 0,45), mientras que el pH mostró una asociación negativa (ß = -1,50; IC_95%_: -3,06 a -0,06), lo que indica un menor desarrollo larvario en aguas más alcalinas.

**Conclusiones.:**

El control biológico con *P. reticulata* mostró potencial para reducir la presencia de larvas. La temperatura y el pH fueron determinantes clave de la presencia de larvas, lo que subraya la necesidad de considerar los factores ambientales en las estrategias de control de vectores.

Dengue is one of the most significant arboviral diseases in Latin America and the Caribbean, with *Aedes aegypti* as its primary vector. In Colombia, dengue is endemic with periodic epidemic peaks. In 2024 ^1^, more than 243 538 cases were reported nationwide, of which the department of Valle del Cauca accounted for one of the highest incidences in the country. The elimination of breeding sites is the most effective control strategy; however, the presence of stormwater drains in urban environments poses a challenge, as they have been identified as key sites for mosquito larval development ^2^^,^^3^.

In Cali, Colombia, local entomological surveillance has shown that stormwater drains contribute substantially to larval densities of *Aedes (A. aegypti* and *A. albopictus),* highlighting their epidemiological importance and the need for sustainable control alternatives. Local entomological studies have demonstrated the coexistence of *Aedes aegypti* and *Culex quinquefasciatus* larvae in storm water drains and other artificial containers in Cali, emphasizing their role as persistent breeding habitats and the need for sustainable control alternatives ^4^.

Chemical control has historically been the primary strategy for reducing vector density. However, the increasing resistance to insecticides and their environmental impact have driven the search for sustainable alternatives ^5^^,^^6^.

One promising approach is biological control using guppy fish, *Poecilia reticulata,* which are efficient predators of mosquito larvae and can thrive in small, stagnant water bodies ^7^. The World Health Organization (WHO) and the Pan American Health Organization (PAHO) have recommended the inclusion of guppy fish within integrated vector management frameworks, especially in domestic and peri-domestic environments ^8^^,^^9^. Successful community-based implementations have shown significant reductions in larval and pupal indices following *P. reticulata* introduction ^10^.

At the *Universidad del Valle* campus (Cali, Colombia), routine entomological monitoring by the institutional vector surveillance program has identified multiple storm water drains positive for mosquito larvae. Given the persistence of these breeding sites despite traditional control measures, this study aimed to evaluate the effectiveness of biological control using *P. reticulata* in reducing the presence of *Aedes (A. aegypti* and *A. albopictus)* and *Culex* sp. larvae in urban stormwater drains at the *Universidad del Valle.*

## Materials and methods

### Study design

This quasi-experimental study was conducted in the stormwater drains of the *Universidad del Valle,* Meléndez campus, in Cali, Colombia. The objective was to evaluate the effectiveness of biological control of *Aedes (A. aegypti* and *A. albopictus)* and *Culex sp.* through the introduction of *P. reticulata,* comparing larval presence in stormwater drains with fish intervention versus those without intervention over a nine-month follow-up period, from October 2022 to June 2023.

Since this study focused on a biological control strategy recommended by the WHO and implemented within the framework of an institutional public health program, approval from an ethics committee was not required. According to the Colombian *Ministerio de Salud y Protección Social,* Resolution 8430 of 1993, the study does not qualify as biomedical research involving human participants or experimental manipulation of animals, as it evaluated an environmental vector control intervention in urban aquatic systems.

In addition, the study complied with the provisions of Law 84 of 1989 (National Animal Protection Statute), which regulates the ethical use of animals in Colombia. Although *P. reticulata* were not naturally present in the storm drains, their introduction was part of a regulated biological control strategy rather than an experimental procedure. The fish were handled following biosafety and welfare principles to avoid stress, injury, or mortality, and no invasive manipulation, confinement, or laboratory testing was performed.

The intervention was conducted under the authorization and supervision of the *Prevención y Control de Enfermedades Transmitidas por Vectores* program of the *Secretaría de Salud del Distrito Especial* of Cali, in accordance with national environmental and sanitary regulations established by the *Ministerio de Salud y Protección Social* and the *Autoridad Nacional de Acuicultura y Pesca* (AUNAP). All activities adhered to applicable public health, environmental, and animal welfare standards.

The activity was carried out following operational and biosafety guidelines established by national authorities, including AUNAP. The intervention complied with applicable environmental and public health regulations and did not involve any procedures that could cause harm or distress to humans or vertebrate animals.

### Eligibility criteria

The study included stormwater drains with stable water retention and documented history of larval positivity for *Aedes (A. aegypti* and *A. albopictus).* Drains with severe obstructions, excessive pollutant accumulation, or conditions that prevented fish survival were excluded, as well as those with a history of frequent desiccation.

### Sampling and allocation

A stratified cluster sampling method was used, defining key environmental criteria: presence of *Aedes* spp. and *Culex* sp. mosquito larvae, solar exposure (high, medium, or low), surrounding vegetation density (high, medium, or low), and proximity to organic matter sources (high or low). Once these clusters were established, stormwater drains were randomly selected within each stratum and assigned to either the intervention or control groups in an approximate 3:1 ratio.

This allocation ratio was chosen to increase the statistical power of the model and the precision of parameter estimation within the intervention group, given the expected variability in larval presence across drains. The control drains were not individually matched to intervention drains by specific characteristics; instead, stratification ensured comparable environmental conditions across groups.

Out of the 229 stormwater drains on campus, 13 met the eligibility criteria and were included in the study; 10 were assigned to the intervention group and three to the control group. During follow-up, one storm drain from the intervention group was excluded due to prolonged desiccation (storm drain 8), resulting in a final total of nine intervention and three control drains. The recruitment of stormwater drains took place in September 2022, while the intervention and monitoring were carried out from October 2022 to June 2023.

Before fish were introduced into the stormwater drains, a pre-monitoring visit was conducted to biologically characterize each drain, determining light intensity, presence or absence of accompanying fauna or flora, and presence or absence of solid and liquid waste. Physicochemical parameters of the water (chlorine, pH, temperature) were measured, and larval density was assessed by counting mosquito larvae by genus and stage. The stormwater drains included in the study were coded and georeferenced, as shown in figure 1.

**Figure 1:**
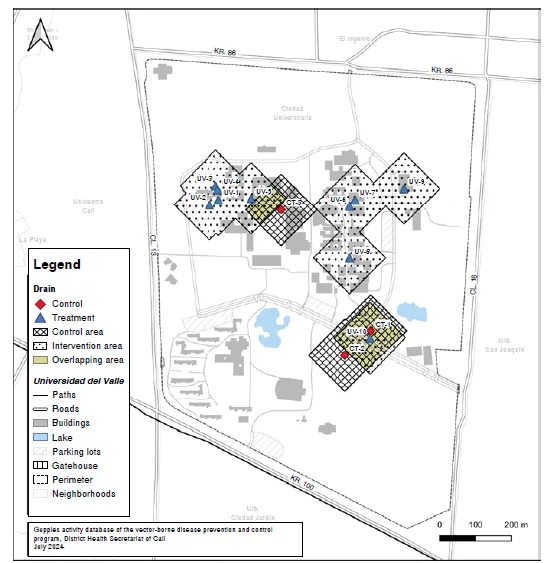
Stormwater drains location

### Intervention

The intervention involved the introduction of *P. reticulata* fish into the stormwater drains selected for the intervention group. Between five and ten individuals were introduced per storm drain, maintaining a ratio of three females per male, with the number of fish adjusted based on the available water volume. The fish were monitored monthly to verify their presence, and in cases where they were not observed during the inspection, restocking was carried out. The stocking, monitoring, and restocking procedures were conducted by trained technical personnel from the *Prevención y Control de Enfermedades Transmitidas por Vectores* program.

The intervention and control groups presented similar environmental conditions at the beginning of the study. The mean water temperature was 23.5 °C (range: 18.4 - 28.7 °C), and the average pH was 7.0 (range: 5.8 -8.2). The initial presence of *Aedes (A. aegypti* and *A. albopictus)* larvae was recorded in 70% (7/10) of the stormwater drains in the intervention group and in 66.7% (2/3) of the control group. The storm drain excluded from the analysis had comparable environmental characteristics to those analyzed, with its exclusion due to prolonged desiccation rather than differences in temperature or pH. No significant differences in temperature or pH were identified between the intervention and control groups (p > 0.05), indicating that both were comparable before the intervention. To account for potential variations, an adjustment was made in the analysis.

### Measurement procedures

Monthly monitoring included the evaluation of *Aedes (A. aegypti* and *A. albopictus)* and *Culex* sp. larvae presence through the visual inspection of four water samples taken with a standardized dipper. Additionally, the presence or absence of fish in the intervention stormwater drains was recorded, and key environmental parameters such as water temperature and pH were measured using a digital thermometer and a calibrated portable pH meter, respectively.

All instruments were verified and calibrated prior to each field campaign according to the manufacturer's specifications and the quality control guidelines established by the Colombian *Instituto de Hidrología, Meteorología y Estudios Ambientales* (IDEAM). Data collection was systematically performed using standardized study forms to ensure consistency and quality in data recording.

### Strategies to minimize bias

To reduce potential bias in the assignment of the intervention, strategies such as stratification by environmental clusters and the use of mixed-effects models were implemented to adjust for repeated measurements within the same stormwater drains, minimizing the impact of clustering. Complete blinding was not possible due to the visibility of the intervention. However, to mitigate bias, the data analysis team operated under a numeric coding scheme to prevent the identification of intervention and control stormwater drains.

### Statistical analysis

The data analysis was conducted in three stages. First, a descriptive analysis was performed, including absolute and relative frequencies of larval presence, as well as measures of central tendency and dispersion for the environmental variables, temperature and pH. Then, the prevalence of *Aedes (A. aegypti* and *A. albopictus)* and *Culex* sp. larvae in each storm drain was estimated, calculating the prevalence ratio as the ratio between the prevalence in each exposed storm drain and the average prevalence across all evaluated stormwater drains.

Subsequently, a Bayesian multilevel logistic regression model was applied to evaluate the relationship between the intervention and larval presence. This model was selected because it appropriately accounts for the hierarchical data structure and the correlation among repeated measurements within the same storm drain. Unlike machine-learning approaches such as random forest or K-means clustering, which are primarily exploratory, multilevel models allow for explicit estimation of group-level variability and inference on fixed effects related to the intervention.

A Bayesian framework was chosen over traditional frequentist models due to the relatively small number of storm water drains and the need to obtain full posterior probability distributions for each parameter, providing more robust uncertainty quantification. The analysis used Markov Chain Monte Carlo sampling via the no-U-turn sampler algorithm. The dependent variable was dichotomous, representing the presence or absence of mosquito larvae in stormwater drains, regardless of genus.

Although both *Aedes (A. aegypti* and *A. albopictus)* and *Culex* sp. larvae were identified during field inspections, they were analyzed jointly as a single outcome variable due to the non-systematic taxonomic differentiation performed in all samples. The variable therefore reflects overall larval presence, with *Aedes (A. aegypti* and *A. albopictus)* considered the primary epidemiological interest given its role as the dengue vector. Explanatory variables included study group (intervention or control), water temperature, and pH. A random intercept was incorporated for stormwater drains to adjust for intragroup correlation arising from repeated measures.

The model was estimated using four parallel chains with 4000 iterations each, of which the first 1000 per chain were designated as warm-up (burn-in), yielding a total of 12 000 post-warm-up samples for posterior inference. Weekly informative priors were applied to the regression coefficients to enhance model stability, and a normal distribution with a mean of 0 and wide variance was used for the intercept.

Model convergence was evaluated through several diagnostics: all parameters reached Rhat values equal to 1.00, and both bulk and tail effective sample sizes exceeded 4000, indicating stable sampling and sufficient chain mixing. Visual inspection of trace plots confirmed that convergence was achieved before the end of the warm-up phase, with no divergent transitions detected. This approach ensured robust estimation of the effects of the intervention and environmental conditions on the presence of larvae in the evaluated stormwater drains.

### Interaction analysis and missing data handling

Interactions between the intervention and environmental variables were assessed using likelihood ratio tests to determine whether temperature and pH significantly modified the effect of the intervention on larval presence. All statistical analyses were conducted using R software (version 4.3.2). A significance level of p < 0.05 was considered for all statistical tests.

## Results

Table 1 presents the descriptive characterization of the stormwater drains evaluated in the study, categorized by storm drain number and summarized overall. The table includes variables related to the intervention (presence of fish and fish restocking), larval counts (total *Aedes -A. aegypti* and *A. albopictus-*and total *Culex* sp.), and environmental parameters (pH and temperature).

**Table 1:** Characteristics of the stormwater drains

Storm drain		I1	I2	I3	I4	I5	I6	I7	I8	I9	C1	C2	C3	Overall
With fish														
	No [n (%)]	7 (50.0%)	2 (14.3%)	2 (14.3%)	0 (0%)	5 (35.7%)	5 (35.7%)	3 (21.4%)	12 (85.7%)	5 (35.7%)	14 (100%)	14 (100%)	14 (100%)	83 (49.4%)
	Yes [n (%)]	7 (50.0%)	12 (85.7%)	12 (85.7%)	14 (100%)	9 (64.3%)	9 (64.3%)	11 (78.6%)	2 (14.3%)	9 (64.3%)	0 (0%)	0 (0%)	0 (0%)	85 (50.6%)
Fish restocking														
	No [n (%)]	7 (50.0%)	12 (85.7%)	12 (85.7%)	14 (100%)	9 (64.3%)	8 (57.1%)	11 (78.6%)	2 (14.3%)	10 (71.4%)	14 (100%)	14 (100%)	14 (100%)	127 (75.6%)
	Yes [n (%)]	7 (50.0%)	2 (14.3%)	2 (14.3%)	0 (0%)	5 (35.7%)	6 (42.9%)	3 (21.4%)	12 (85.7%)	4 (28.6%)	0 (0%)	0 (0%)	0 (0%)	41 (24.4%)
Total *Aedes*														
	Mean (SD)	8.36 (18.6)	1.36 (4.53)	0 (0)	2.14 (8.02)	3.43 (9.27)	1.36 (5.08)	0.0714 (0.267)	0.357 (0.497)	0 (0)	0 (0)	0 (0)	0.643 (1.45)	1.48 (6.91)
	Median	0 [0, 67.0]	0 [0, 17.0]	0 [0, 0]	0 [0, 30.0]	0 [0, 35.0]	0 [0, 19.0]	0 [0, 1.00]	0 [0, 1.00]	0 [0, 0]	0 [0, 0]	0 [0, 0]	0 [0, 4.00]	0 [0, 67.0]
	[min., max.]													
Total *Culex*														
	Mean (SD)	0.571 (1.28)	1.43 (4.55)	0.357 (1.34)	0.429 (0.756)	9.71 (27.1)	2.21 (6.47)	0.929 (2.43)	3.93 (11.6)	0.286 (0.611)	6.50 (9.97)	2.14 (2.21)	1.43 (2.53)	2.49 (9.47)
	Median	0 [0, 4.00]	0 [0, 17.0]	0 [0, 5.00]	0 [0, 2.00]	0 [0, 100]	0 [0, 24.0]	0 [0, 8.00]	0 [0, 44.0]	0 [0, 2.00]	0.500 [0, 31.0]	2.00 [0, 6.00]	0 [0, 7.00]	0 [0, 100]
	[min., max.] pH													
pH														
	Mean (SD)	7.01 (0.274)	7.11 (0.170)	7.04 (0.295)	6.97 (0.249)	7.10 (0.204	7.03 (0.205)	6.94 (0.191)	7.31 (0.268)	6.96 (0.386)	6.99 (0.388)	6.89 (0.170)	6.86 (0.145)	7.02 (0.275)
	Median	6.80	7.20	6.80	6.80	7.20	7.20	6.80	7.50	6.80	7.20	6.80	6.80	6.80
	[min., max.]	[6.80, 7.50]	[6.80, 7.20]	[6.80, 7.50]	[6.80, 7.50]	[6.80, 7.40]	[6.80, 7.20]	[6.80, 7.20]	[6.80, 7.50]	[6.80, 8.20]	[5.80, 7.20]	[6.80, 7.20]	[6.80, 7.20]	[5.80, 8.20]
Temperature														
	Mean (SD)	24.2 (1.55)	21.2 (1.22)	21.6 (1.11)	22.5 (2.34)	21.7 (1.16)	23.4 (0.947)	24.2 (1.71)	23.7 (1.94)	25.3 (2.35)	24.4 (2.15)	24.6 (2.14)	22.3 (2.17)	23.3 (2.18)
	Median	23.4	21.2	21.7	21.8	21.0	23.4	24.3	23.1	24.7	23.3	24.1	21.8	23.2
	[min., max.]	[22.6, 27.4]	[18.3, 23.2]	[18.4, 23.2]	[20.1, 27.0]	[20.7, 23.9]	[21.5, 25.2]	[20.8, 27.0]	[21.0, 27.3]	[21.6, 30.8]	[21.0, 28.7]	[21.2, 29.0]	[18.4, 28.0]	[18.3, 30.8]

C1-C3: control storm drains; I1-I9: intervention storm drains containing *Poecilia reticulata;* with fish: number and percentage of inspections where fish were absent; fish restocking: storm drains where fish were reintroduced after disappearance; total *Aedes* spp.: total count of *Aedes* spp. (A. *aegypti* and *A. albopictus)* larvae observed per drain; total *Culex* sp: total count of *Culex* sp. larvae observed per drain

Regarding the intervention, 49.4% (83/168) of the stormwater drains were classified as having the presence of fish (P. *reticulata),* while 50.6% (83/168) did not contain fish during the evaluation. Additionally, in 75.6% (127/168) of the cases, at least one fish restocking was performed, reflecting the need for continuous maintenance in some stormwater drains to ensure the fish's presence.

Larval counts were highly heterogeneous across stormwater drains. Six stormwater drains concentrated the highest larval counts, whereas the remaining six showed counts close to zero.

Concerning environmental parameters, the average pH of the stormwater drains was 7.02 (SD = 0.275), with minimal variability among them. The mean water temperature was 23.2 °C (SD = 2.18), with values ranging between 18.3 and 30.8 °C. Temperature variations were observed across different stormwater drains, which could influence the survival of both mosquito larvae and the fish used in the intervention.

Figure 2 shows the prevalence ratio of larvae per storm drain, comparing the prevalence of each storm drain with the overall average across all evaluated drains. A prevalence ratio greater than 1 indicates that the drain presented a higher prevalence of larvae compared to the average, while values below 1 indicate a lower prevalence. The analysis considered the total larval count, including both *Aedes (A. aegypti* and *A. albopictus)* and *Culex* sp., without species differentiation, as identification at the larval stage was not systematically conducted in all samples. The black dashed line represents the reference prevalence ratio of 1. It was observed that six stormwater drains exhibited prevalence ratios above the overall average (PR > 1), indicating greater larval persistence, whereas the remaining six had prevalence ratios below the overall average (PR < 1). In the control group, all three stormwater drains showed a prevalence ratio up to 1.5 times higher than the overall average.

**Figure 2:**
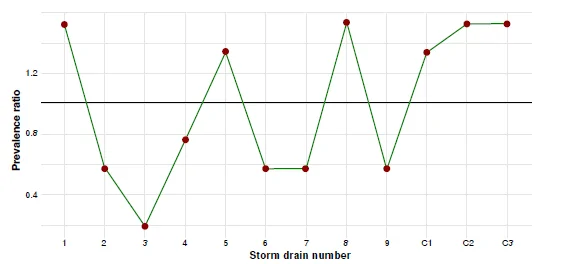
Prevalence ratio by stormwater drains

Table 2 presents the estimated coefficients of the Bayesian multilevel logistic regression model used to assess the relationship between larval presence and the independent variables: intervention group (intervention versus control), temperature, and pH. A random effect term was included for storm drain number to adjust for the hierarchical structure of the data due to repeated measurements within the same drains over time.

**Table 2:** Bayesian multilevel logistic regression model

Parameters	Estimation	SE	IL 95% CI	SL 95% CI	Rhat	Bulk ESS	Tail ESS
Intercept	4.74	5.47	-5.65	15.53	1	7478	6239
Intervention group	-0.9	0.57	-2.01	0.21	1	4775	4799
Temperature	0.25	0.1	0.07	0.45	1	6102	5358
pH	-1.5	0.77	-3.06	-0.06	1	6009	5385

SE: standard error; IL: inner limits; CI: confidence interval; SL: superior limits; ESS: effective sample size

The intercept represents the baseline probability of larval presence in the reference group (control drains) under average temperature and pH conditions. Its estimate (ß = 4.74, 95% CI: -5.65 to 15.53) shows a wide credible interval, reflecting high uncertainty in the baseline level of larval prevalence, likely explained by the environmental heterogeneity and limited number of observations.

For the intervention group, the posterior mean coefficient was negative (ß = -0.90, 95% CI: -2.01 to 0.21), corresponding to an odds ratio (OR) of approximately 0.41 (95% CI: 0.13 to 1.23). This suggests a trend toward reduced larval presence in storm drains containing *P. reticulata* compared to controls, although the credible interval includes 1, indicating that this effect is not conclusive in the Bayesian sense.

Temperature showed a positive association with larval presence (ß = 0.25; OR = 1.28; 95% CI: 1.07 to 1.57), meaning that each 1 °C increase in temperature raised the odds of larval occurrence by about 28%. Conversely, pH had a negative association (ß = -1.50; OR = 0.22; 95% CI: 0.05 to 0.94), indicating that more alkaline conditions significantly decreased the probability of larval presence.

The model's hyperparameters indicated moderate variability among storm drains, with a standard deviation of 0.60 for the intercept, supporting the inclusion of a multilevel structure to account for intrinsic differences between drains. All Rhat values were equal to 1.00, confirming that the model achieved convergence and that posterior estimates were stable across chains.

Overall, these results highlight that environmental factors -particularly temperature and pH- play a crucial role in larval development, while the observed trend in the intervention group supports the potential of *P. reticulata* as a biological control agent under certain ecological conditions. The strong inverse association between pH and larval presence provides novel evidence on the physicochemical constraints influencing mosquito breeding in urban storm drains.

Figure 3 shows the posterior distributions (left) and trace plots (right) of the estimated parameters in the Bayesian multilevel logistic regression model. The histograms reflect the uncertainty of the estimates, while the trace plots indicate adequate convergence of the Markov chains.

**Figure 3:**
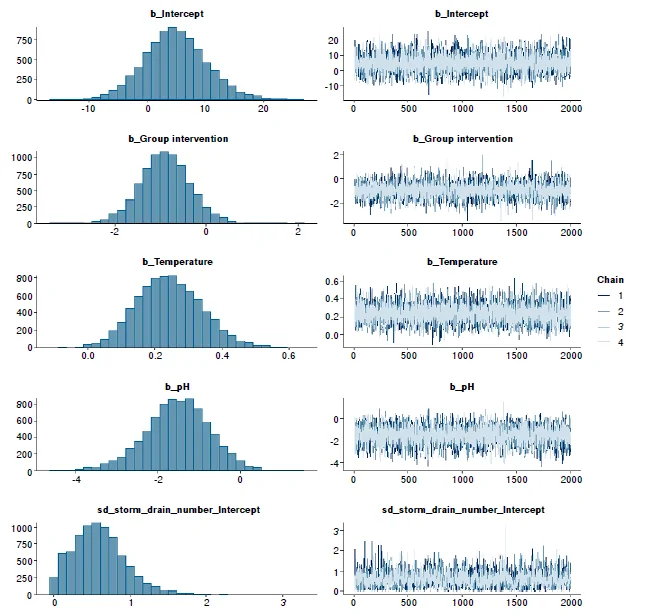
Posterior distribution and trace plots of the estimated parameters in the multilevel model

The coefficient for the intervention group was centered around negative values, suggesting a lower presence of larvae in stormwater drains with *P. reticulata.* Temperature shows a positive association with larval presence, while pH is negatively associated. The variance between stormwater drains justifies the inclusion of a random effect. These results reinforce the validity of the model and suggest that the fish-based intervention reduces larval presence, influenced by environmental factors.

No additional analyses were conducted, as the study was not designed with sufficient statistical power to evaluate additional hypotheses or specific subgroups. However, interactions between the intervention and environmental conditions were explored, but no significant effects were found to justify further analysis.

## Discussion

This study evaluated the use of *P. reticulata* as a biological control strategy to reduce *Aedes (A. aegypti* and *A. albopictus)* and *Culex* sp. larvae in urban stormwater drains. While the results suggest a potential reduction in larval presence in the drains where fish were introduced, the estimated effect for the intervention group was inconclusive, as the credibility interval of the Bayesian model included the null value. This indicates that although there was a trend toward larval reduction in the intervention drains, the evidence was not strong enough to confirm a significant effect.

The analysis using a Bayesian multilevel model allowed adjustment for the hierarchical structure of the data, capturing intra-drain variability generated by repeated measurements over time. The main advantage of this approach was the incorporation of prior distributions that stabilized the estimates under the small-sample context, reducing uncertainty compared to standard frequentist methods ^11^. However, beyond the statistical adjustment, the stratified cluster design also revealed important ecological patterns.

Drains located in areas with higher sunlight exposure and greater vegetation density tended to maintain higher larval prevalence, suggesting that microenvironmental conditions such as temperature, organic matter, and shade play a crucial role in sustaining mosquito breeding sites even under biological control interventions. These findings emphasize the relevance of including stratification variables not only for sampling design but also for ecological interpretation of intervention outcomes.

Biological control with *P. reticulata* relies on its ability to prey on mosquito larvae. However, the results were inconclusive, which may be due to multiple factors. Among them, the presence of *Culex* sp. -whose larvae tend to remain deeper in the water column and are less exposed to surface predation- may have reduced the effectiveness of fish predation compared with *A. aegypti.*

Additionally, environmental variations in temperature and pH could have influenced the predatory activity of the fish ^12^. Specifically, pH and temperature were identified as significant variables in the model, suggesting that environmental conditions played a key role in the ecosystem dynamics studied.

Previous studies have shown that pH can influence osmotic regulation and the behavior of *P. reticulata*^13^, while the optimal temperature for the development of *A. aegypti* ranges between 24 and 32 °C, favoring their proliferation within this range ^14^.

An important limitation of this study is that larval counts were analyzed jointly for *Aedes (A. aegypti* and *A. albopictus)* and *Culex* sp. without systematic taxonomic differentiation. Although both genera share similar aquatic habitats in urban stormwater drains, *A. aegypti* is the epidemiologically relevant vector for dengue transmission, while *Culex* sp. are generally nuisance species with different ecological dynamics. Consequently, the lack of species-level separation may have introduced non-differential misclassification in larval prevalence estimates, potentially attenuating the observed association between the intervention and the reduction of *Aedes* spp. larvae. Future studies should incorporate genus or species level identification to strengthen ecological and epidemiological interpretation of biological control interventions.

From an ecological perspective, the survival and reproduction of *P. reticulata* in mosquito breeding sites may have been affected by factors such as water quality and the presence of other predators. *P. reticulata* is a freshwater species sensitive to physicochemical changes, which could have influenced its survival and, consequently, its predatory capacity on mosquito larvae.

Similar findings have been reported in other urban settings: studies conducted in Cuba, Honduras, and Cambodia observed that variations in water temperature, organic matter, and oxygen availability significantly affect guppy survival and larval predation efficiency in stormwater drains and domestic containers ^15^. In urban environments, stormwater drains often present fluctuations in pollutant concentration and dissolved oxygen, factors that can negatively affect the viability of these fish as biological control agents.

From an operational perspective, the intervention was successfully implemented by technical staff from the *Prevención y Control de Enfermedades Transmitidas por Vectores* program, demonstrating its feasibility in a public health context ^16^. However, the need for continuous monitoring and periodic fish reintroduction may represent a logistical barrier to large-scale implementation. Similar operational challenges have been described in other experiences with biological control programs, which emphasize the importance of integrating this strategy with complementary interventions such as regular drain cleaning, removal of organic debris, targeted larvicide in high-risk areas, and community participation programs to maintain storm drain functionality and prevent recolonization by mosquitoes ^7^. These combined approaches have shown greater sustainability and long-term effectiveness in reducing vector densities in urban environments.

Overall, the stratified cluster design provided valuable insight into environmental heterogeneity and its influence on larval persistence. The combination of stratified sampling and hierarchical modeling enabled the identification of key ecological determinants that modulate the success of biological control strategies.

Future studies should explore integrating hierarchical models with spatial or network-based approaches to better represent the ecological dependencies across stormwater drains within the same urban catchment and to evaluate the synergistic effects of combining biological, environmental, and community-based interventions.
